# Effect of a Digital Health Exercise Program on the Intention for Spinal Surgery in Adult Spinal Deformity: Exploratory Cross-Sectional Survey

**DOI:** 10.2196/66889

**Published:** 2025-04-29

**Authors:** Marsalis Christian Brown, Christopher Quincy Lin, Christopher Jin, Matthew Rohde, Brett Rocos, Jonathan Belding, Barrett I Woods, Stacey J Ackerman

**Affiliations:** 1 Department of Orthopaedic Surgery, MetroHealth Medical Center, 2500 MetroHealth Dr, Cleveland, OH, 44109, United States, 1 614-441-3703; 2 Department of Medicine, The Larner College of Medicine, The University of Vermont, Burlington, VT, United States; 3 Department of Medical, Medical College of Wisconsin, Milwaukee, WI, United States; 4 The Department of Orthopaedics, Zucker School of Medicine at Hofstra/Northwell, Hempstead, NY, United States; 5 Duke Spine Center, Duke University Health System, Durham, NC, United States; 6 Department of Spine Surgery, Rothman Orthopaedic Institute, Thomas Jefferson University Rothman Institute, Philadelphia, PA, United States; 7 Department of Biomedical Engineering, Johns Hopkins University, San Diego, CA, United States

**Keywords:** adult spinal deformity, scoliosis, nonoperative management, spinal realignment therapy, digital health, program evaluation

## Abstract

**Background:**

Adult spinal deformity (ASD) is a prevalent condition estimated at 38%. Symptomatic ASD is associated with substantial health care costs. The role of nonoperative interventions in the management of ASD remains elusive. The National Scoliosis Clinic’s (NSC) scoliosis realignment therapy (SRT) is a personalized digital health exercise program for the nonoperative management of ASD.

**Objective:**

This exploratory study had two objectives: (1) to evaluate the effect of the SRT program on users’ intention of having spinal fusion; and (2) from a US payer perspective, to estimate the annual cost savings per 100,000 beneficiaries by averting spinal surgery.

**Methods:**

Individuals were enrolled in the SRT study from October 1, 2023 to September 1, 2024. Participants completed a web-based, cross-sectional survey about their history of prior scoliosis surgery and intent of having surgery before and after use of SRT (on a 4-point Likert scale, where 1 = “No Intent for Surgery” and 4 = “High Intent for Surgery”). Intent for surgery before and after participation in SRT was compared using a nonparametric Wilcoxon signed-rank test for paired data. Annual cost savings per 100,000 beneficiaries by averting spinal fusions were estimated separately for commercial payers and Medicare using published literature and public data sources. Payer expenditures were inflation-adjusted to 2024 US dollars using the Hospital Services component of the Consumer Price Index.

**Results:**

A total of 62 NSC members (38.8%) responded to the survey and were enrolled in the SRT program for an average (SD) of 17 (12) weeks. The mean (SD) age was 65.3 (13.5) years, and the majority were female (47/48, 98%) and White (45/46, 98%). Among the SRT users who did not have prior scoliosis surgery (n=56), 14% (8/56) reported a decrease in intent for surgery (that is, a lower Likert score) with the use of SRT. The mean (SD) intent for surgery scores before compared to after SRT were 1.29 (0.53) and 1.14 (0.35), respectively (mean difference 0.15 [*P*=.006]). Participants with “No Intent for Surgery” pre- versus postuse of SRT (42/56 versus 48/56, respectively) corresponded to an absolute risk reduction of 11% and a number needed to treat of 9 to avert one spinal fusion. Among the 6 participants who transitioned to “No Intent” for spinal surgery with the use of SRT, 3 were aged <65 years and 3 were ≥65 years of age. The annual cost savings from averted spinal surgeries were estimated at US $415,000 per 100,000 commercially-insured beneficiaries and US $617,000 per 100,000 Medicare beneficiaries.

**Conclusions:**

SRT is a personalized, scoliosis-specific digital health exercise program with the potential for averting 1 spinal surgery for every 9 participants, resulting in a substantial reduction in payer expenditures while improving the quality of care for commercial payers and Medicare beneficiaries.

## Introduction

Adult spinal deformity (ASD) is a common condition with an estimated prevalence of 38% for primary (de novo) degenerative scoliosis [1,2]. ASD is a 3-dimensional deformity of the spine defined by a major curve magnitude angle of ≥10°, and women are more likely to have the diagnosis [1-4]. Patients with symptomatic ASD report increased back pain as well as lower health-related quality of life as demonstrated by worse SF-36 (36-Item Short Form Health Survey) scores compared to the general population [5,6].

As the US population continues to age, there is growing interest in understanding the most effective means to manage ASD. While nonoperative modalities are typically the first-line treatment for patients with symptomatic ASD, the role of nonoperative management has been questioned, with some studies reporting 2-year costs ranging from US $2041 to US $14,022 without improvements in patient outcomes [7-9]. In contrast, several studies have reported that patients who receive operative interventions for ASD have significantly reduced disability and pain and better improvement in clinical outcomes compared to nonoperative treatment [9-12]. However, spinal fusion is costly (Medicare reimbursement of $60,269 [13]) and carries significant risks; a multicenter database study of nearly 1000 adult surgical patients with ASD with 2-year follow-up reported an overall complication rate of 67.4% [14], and a separate meta-analysis demonstrated complication rates ranging from 17.0% to 71.5% [12]. Considering the substantial economic burden and complication rate associated with operative interventions for ASD—and noting that, other digital care programs in orthopedics (low back pain and chronic knee pain) have demonstrated significant reductions in surgical intent [15-17]—this study sought to further elucidate the role of a digital health program in the nonoperative management of ASD.

The National Scoliosis Clinic’s (NSC) Scoliosis Realignment Therapy (SRT) is a remote, exercise-based, ScolioPilates® therapy specifically designed for individuals with scoliosis. ScolioPilates® is a scoliosis-specific exercise program using elongation, corrective breathing, strengthening, and activities of daily living to address pain associated with scoliosis. Additionally, members of SRT are enrolled in a supportive community with virtual group sessions, education, and AI-driven technology to assess spinal curvature with personalized therapy. While Clohisy et al [18] reported that the majority of patients crossover from nonoperative to operative treatment due to perceived worsening of symptoms, Rhode et al [19] recently reported that the use of SRT for 6 weeks resulted in significant improvement in pain as measured using the Scoliosis Research Society Health-Related Quality of Life Questionnaire (SRS-22r).

This exploratory study had two objectives: (1) to evaluate the effect of the SRT program on users’ intention of having spinal fusion; and (2) from a US payer perspective, to estimate the annual cost savings per 100,000 beneficiaries by averting spinal surgery.

## Methods

### Recruitment

Individuals with scoliosis were recruited to the NSC through multiple mechanisms, including social media and the NSC website. NSC members were enrolled nationwide in the SRT study between October 1, 2023 and September 1, 2024. All NSC members using SRT during the aforementioned time period were invited by email to participate in the closed, web-based, cross-sectional survey about surgical intention. A formal sample size calculation was not performed, given that this was an exploratory study. In addition to demographics and clinical characteristics, the survey asked, “Has a surgeon ever told you you need scoliosis surgery, or offered you surgery?”, “Have you ever intended to have scoliosis surgery?” and “Since you have started working with the NSC SRT, what is your current intention to have scoliosis surgery?” Responses to the questions about surgical intent were graded on a 4-point Likert scale, where 1=No Intent for Surgery, 2=Low Intent, 3=Moderate Intent, and 4=High Intent for Surgery. Given that this was an exploratory web-based survey, radiographic parameters were not collected.

### Statistical Analysis

The primary outcome measure was the intent to pursue surgical correction of scoliosis pain. A nonparametric Wilcoxon signed-rank test for paired data was conducted to evaluate the difference in intention scores before and after SRT. The Wilcoxon test is appropriate for small samples that are not normally distributed. All analyses were performed using R software (version 4.4.1; R Foundation for Statistical Computing).

For potentially averted spinal surgeries, annual cost savings per 100,000 beneficiaries were estimated separately for US commercial payers and Medicare using published literature and multiple public data sources (Table 1). Payer expenditures were inflation-adjusted to US dollars in 2024 by using the Hospital Services component of the Consumer Price Index.

**Table 1.** Parameter values, data sources, and estimated cost savings from potentially averted spinal fusions among individuals with adult spinal deformity in the United States (2024, $US) [20].

Parameter	Value		Source
	Commercial	Medicare	
Medical resource use for scoliosis by adults, %	0.502	1.531	[20,21]
Adult scoliosis patients per 100,000 beneficiaries, n	502	1531	Derived
Convert from nonoperative care to spinal fusion (annual), %	21.7	21.7	[22]
Conversions to spinal fusion (annual), n	109	332	Derived
Absolute risk reduction in intent for surgery, %	11	11	SRT survey
SRT^a^ participation rate (users), %	28	28	[23]
Spinal fusions averted annually per 100,000 beneficiaries, n	3.4	10.2	Derived
Spinal fusion insurer payment, US $^b^^c^	123,551^d^	60,269	[13,24,25]
Annual cost savings per 100,000 beneficiaries, $	414,534	616,504	Derived

a SRT: scoliosis realignment therapy.

b Weighted average of diagnosis-related group (DRG) 456 (spinal fusion with spinal curvature with major complication, 25%), DRG 457 (spinal fusion with spinal curvature with complication, 59%), and DRG 458 (spinal fusion with spinal curvature without complication or major complication, 16%).

c Inflated from 2022 to 2024 (half) US $ using the Consumer Price Index for hospital services; multiplier 1.097.

d Calculated using a Medicare-to-commercial payment multiplier of 2.05 for hospital inpatient services.

### Data Exclusion

Users with prior spinal fusion surgery were excluded from the surgical intent analysis. Participants were given 2 weeks to respond to the survey, with subsequent reminders. Users who failed to respond to the surgical intent questions were excluded.

### Ethical Considerations

This web-based survey evaluated a wellness program, not a medical treatment, and study data were deidentified for privacy and confidentiality protection. The datasets generated and analyzed during this study were restricted to the researchers conducting the analysis. NSC members who participated in the web-based survey study were provided US $50 gift cards for the completion of the survey.

Per the Office of Human Research Protections, under the US Department of Health and Human Services, research that (1) involves only survey procedures of adults and (2) is collected in a deidentified fashion is exempt from institutional review board (IRB) review and informed consent can be waived [26]. A retrospective IRB exemption was obtained for this study, confirming that both conditions are met, and therefore, this study was IRB exempt, and informed consent was not indicated.

## Results

### User Statistics

In total, 160 NSC members were invited to participate in the survey; 62 (38.8%) individuals nationwide voluntarily completed the survey. Six participants were excluded from the intention to pursue surgery analysis: 5 who had prior fusion surgery and 1 who did not respond. Among survey respondents who reported age (N=47), the mean (SD) age was 65.3 (13.5) years (age <65 years, 40%; age ≥65 years, 60%). The mean (SD) SRT duration of use was 118 (86.6 days); the majority were female (47/48, 98%) and White (45/46, 98%; Table 2).

**Table 2.** Demographics and clinical characteristics of scoliosis realignment therapy (SRT) cross-sectional survey participants with adult spinal deformity (n=62).

Characteristic	Value
Age in years, mean (SD)	65.3 (13.5)
Age <65 years, n (%)	19 (30.6)
Age ≥65 years, n (%)	28 (45.2)
Did not respond	15 (24.2)
Gender, n (%)	
Female	47 (75.8)
Male	1 (1.6)
Did not respond	14 (22.6)
Race, n (%)
White	45 (72.6)
American Indian and Caucasian	1 (1.6)
Did not respond	16 (25.8)
SRT Duration of Use in days, mean (SD)	118 (86.6)
Offered scoliosis surgery in the past, n (%)	
Yes	15 (24.2)
No	26 (41.9)
Did not respond	21 (33.9)
Prior fusion surgery for scoliosis, n (%)^a^	
Yes	5 (8.1)
No	56 (90.3)
Did not respond	1 (1.6)

a Individuals with either prior fusion surgery or no response excluded from surgical intent analysis.

### Evaluation Outcomes

#### Surgical Intent

Overall, 14% of participants (8/56) reported a decrease in intent for scoliosis surgery (ie, a lower Likert score) with the use of SRT, and no participants reported an increase in surgical intent. The mean (SD) intent for surgery scores before compared to after SRT were 1.29 (0.53) and 1.14 (0.35), respectively (mean difference 0.15 [*P*=.006]; Table 3). Participants with “No Intent” for spinal surgery pre- versus postuse of SRT (42/56 versus 48/56, respectively) corresponded to an absolute risk reduction of 11% and a number needed to treat of 9 to potentially avert 1 spinal fusion (1 divided by 0.11). Among the 6 participants who transitioned to “No Intent” for spinal surgery with the use of SRT, 3 were aged <65 years (ages 30, 62, and 64 years, respectively) and 3 were aged ≥65 years (ages 73, 73, and 82 years, respectively).

**Table 3.** Intentions for surgical intervention for adult spinal deformity prior to and after using scoliosis realignment therapy (SRT; cross-sectional survey; n=56).

Statement	Percentage of respondents				Mean (SD)^a^	
	No intent	Low	Moderate	High		
Have you ever intended to have scoliosis surgery?	75.0	21.4	3.6	0.0	1.29 (0.530)	
Since you have started working with the NSC^b^ SRT, what is your current intention to have scoliosis surgery?	85.7	14.3	0.0	0.0	1.14 (0.353)	

a Four-point Likert scale, where 1=No Intent for Surgery, 2=Low Intent, 3=Moderate Intent, and 4=High Intent. *P*=0.006, Wilcoxon signed-rank test.

b NSC: National Scoliosis Clinic.

#### Estimated Cost Savings by Averting Spinal Fusion

The percentage of adult patients using medical services for scoliosis was 0.502% (age 18‐64 years) and 1.531% (age ≥65 years) for commercial and Medicare beneficiaries, respectively, or, 502 and 1531 per 100,000 beneficiaries, respectively (Table 1). An estimated 21.7% (41/189) of these patients converted from nonoperative care to spinal fusion annually. Applying the SRT-related 11% absolute risk reduction and an assumed 28% SRT participation rate based on digital health wellness intervention programs without incentives, a total of 3.4 fusions would potentially be averted annually per 100,000 commercially insured beneficiaries and 10.2 fusions potentially avoided annually per 100,000 Medicare beneficiaries. Applying a Medicare payment of US $60,269 for hospital inpatient spinal fusion (weighted average of diagnosis-related groups [DRGs] 456‐458 in 2024 in US $) and multiplier for Medicare-to-commercial payment of 2.05 (ie, commercial payment of US $123,551), the annual cost savings from averted surgeries were estimated at US $415,000 per 100,000 commercially insured beneficiaries, and US $617,000 per 100,000 Medicare beneficiaries (Table 1).

## Discussion

### Principal Findings

This web-based, cross-sectional survey study explored whether the use of SRT, a personalized digital therapy program for adults with scoliosis, resulted in a reduction in intent for spinal surgery. After using SRT for an average of 17 weeks, an 11% absolute risk reduction in intent for surgery was observed, which corresponds to a number needed to treat of 9, suggesting that one spine surgery could potentially be averted for every 9 individuals using SRT. Hence, SRT offers a promising nonoperative therapy that may decrease spinal fusions with its associated risks and economic burden.

### Comparison With Prior Work

The reduced intent in pursuing surgery with the use of SRT is consistent with other digital care programs in orthopedics, recognizing that scoliosis is a unique condition with its own clinical challenges. For a 12-week digital care program in low back pain, Shebib et al [15] demonstrated a significant reduction in surgical interest (*P*=.01). Similarly, Smittenaar and colleagues [16] reported a decrease in surgery intent at 3 months (*P*<.001) with a 12-week digital care program for chronic knee pain. Mecklenburg et al [17] also found that the self-reported likelihood of having surgery decreased over 1 year with the use of a digital care program for chronic knee pain (*P*=.01).

Investigators have reported conversion rates from nonoperative to operative treatment ranging from 12.7% (24/189) within 1 year to 31% (42/135) after 6 months [18,22]. Clohisy et al [18] further noted that the majority (90%) of patients crossed over from nonoperative to operative treatment due to perceived worsening of symptoms, and the remainder because the patients believed that the nonsurgical therapies were ineffective. Rhode et al [19] recently reported outcomes following 6 weeks of SRT using SRS-22r. Specifically, a significant improvement was observed in the pain (*P*<.001), self-image (*P*=.05), and mental health (*P*<.001) subdomains of the SRS-22r, with the improvement in the pain subdomain exceeding the minimal clinically important difference threshold. In addition, these investigators reported high satisfaction with the SRT program (9.5 out of 10, where 1=Extremely Dissatisfied and 10=Extremely Satisfied) [19]. Given that the SRT cohort reported in the study by Rhode et al (n=23) [19] is a subset of those reported herein (n=62), the statistically significant decrease in intent for surgery is consistent with the previously reported perceivable improvement in pain with the use of SRT. These consistent findings across SRT studies support the validity of using the question on surgical intent to extrapolate to the estimated cost savings.

In light of the aging population among other factors, Wadhwa and colleagues [27] reported that, among both commercial and Medicare beneficiaries, the rate of fusion for adult spinal deformity surgery doubled from 2007 to 2015. In addition, Passias et al [9] reported that the mean 2-year cost of nonoperative treatment was US $2041, and the mean 2-year cost for operative treatment was US $66,860 based on Medicare reimbursements. As such, the estimated cost savings for averted surgeries reported in the present study are likely underestimated for multiple reasons, specifically, the percentage of beneficiaries seeking medical care for scoliosis is based on data from 2013 [20], the DRG reimbursement amounts reflect only the spinal surgery hospitalization (including the global period) [13], and incentives could increase the SRT participation rate [23].

The cost analyses are based on the best available published data, recognizing that more recent trends in insurer reimbursement and technological advancements may not be reflected. The cost-savings estimates rely on several assumptions, including a 28% participation rate for intervention-focused digital wellness programs. Mattke et al [23] described the relationship between incentive structure and participation rate, noting that the use of incentives increased wellness program participation. Our analysis assumed no incentives with an intervention-focused participation rate of 28% (versus 30% if any incentives). Others have reported a 32% participation rate for physical well-being programs [28]. Hence, assuming the absence of incentives offers a conservative estimate of the participation rate.

### Limitations

There are several limitations of this formative research study. First, participants used SRT for an average of 17 weeks, which begs the question about whether the observed decrease in surgical intent would be sustained. That said, Smittenaar et al [16] found that the reduction in surgery interest continued for 6 months following the initiation of a digital health program for chronic knee pain (*P*<.001), and Mecklenburg et al [17] reported that the decreased likelihood of knee surgery with the use of a digital care program was sustained over 5 years (*P*=.002). Second, the self-reported survey data are subjective and participants completed both the pre- and post-SRT questions about surgical intention after initiating SRT, which potentially may be subject to recall bias. Third, the pre-post study design may be subject to confounding because it lacks an independent control or comparison group and is less rigorous than a randomized control trial; notwithstanding, each participant served as their own control. Furthermore, study participants were not blinded in that they were aware of using SRT, which may have introduced the possibility of performance bias. Lastly, although participants represented a nationwide sample of adults with scoliosis, the study included a relatively small cohort that did not permit the exploration of nuanced effects and was not ethnically diverse; therefore, the results may not be generalizable. Future studies will explore the durability of SRT use on surgical intent with a larger, more diverse cohort.

### Conclusions

SRT is a personalized, scoliosis-specific digital health exercise program with the potential for averting 1 spinal fusion for every 9 participants, resulting in a substantial reduction in payer expenditures while improving the quality of care for commercial payer and Medicare beneficiaries. The SRT remote, digital care program holds promise as a feasible strategy to transform the nonoperative paradigm in adult spinal deformity. Digital care delivery is becoming more common with currently available health technology, and can increase patient agency and engagement, while improving overall outcomes and decreasing health care spending.

## Abbreviations

ASD: adult spinal deformity
DRG: diagnosis-related group
IRB: institutional review board
NSC: National Scoliosis Clinic
SF-36: 36-Item Short Form Health Survey
SRT: scoliosis realignment therapy
